# The *Phaseolus vulgaris PvTRX1h* gene regulates plant hormone biosynthesis in embryogenic callus from common bean

**DOI:** 10.3389/fpls.2015.00577

**Published:** 2015-07-28

**Authors:** Aarón Barraza, José L. Cabrera-Ponce, Roberto Gamboa-Becerra, Francisco Luna-Martínez, Robert Winkler, Raúl Álvarez-Venegas

**Affiliations:** Centro de Investigación y de Estudios Avanzados del Instituto Politécnico Nacional, Unidad IrapuatoGuanajuato, México

**Keywords:** *Phaseolus vulgaris*, plant hormone, histone methyltransferase, auxin, callus, somatic embryo

## Abstract

Common bean is the most important grain legume in the human diet. Bean improvement efforts have been focused on classical breeding techniques because bean is recalcitrant to both somatic embryogenesis and *in vitro* regeneration. This study was undertaken to better understand the process of somatic embryogenesis in the common bean. We focused on the mechanisms by which somatic embryogenesis in plants is regulated and the interaction of these mechanisms with plant hormones. Specifically, we examined the role of the gene *PvTRX1h*, an ortholog of a major known histone lysine methyltransferase in plants, in somatic embryo generation. Given the problems with regeneration and transformation, we chose to develop and use regeneration-competent callus that could be successively transformed. Embryogenic calli of common bean were generated and transformed with the PvTRX1hRiA construction to down-regulate, by RNA interference, expression of the *PvTRX1h* gene. Plant hormone content was measured by mass spectrometry and gene expression was assessed by q-PCR. Detailed histological analysis was performed on selected transgenic embryogenic calli. It was determined that down-regulation of *PvTRX1h* gene was accompanied by altered concentrations of plant hormones in the calli. PvTRX1h regulated the expression of genes involved in auxin biosynthesis and embryogenic calli in which *PvTRX1h* was down-regulated were capable of differentiation into somatic embryos. Also, down-regulation of *PvTRX1h* showed increased transcript abundance of a gene coding for a second histone lysine methyltransferase, *PvASHH2h*. Accordingly, the *PvTRX1h* gene is involved in the synthesis of plant hormones in common bean callus. These results shed light on the crosstalk among histone methyltransferases and plant hormone signaling and on gene regulation during somatic embryo generation.

## Introduction

Epigenetic phenomena affect the structure and organization of chromatin and through this mechanism can influence gene expression by modulating the access of regulatory proteins and protein complexes to the genome. Epigenetic mechanisms appear to be involved in almost every aspect of plant life, from embryo development to plant immunity. The former is, in part, apparent as epigenetic mechanisms modulate embryogenic capacity of plant cells in culture (Rival et al., 2013).

Plant hormones are another important regulator of development. They are directly involved in seed germination, tissue and organ differentiation and development, flowering, fruiting, tropisms, and responses to biotic and abiotic stress (Santner and Estelle, 2009). Plant hormones are a structurally unrelated collection of small molecules that act at low concentrations. The main plant hormones and hormone classes are auxins, cytokinins, gibberellins (GA), abscisic acid (ABA), ethylene, brassinosteroids (BR), jasmonic acid (JA), salicylic acid (SA), nitric oxide, and strigolactones.

From this brief review, it should be apparent that both of these areas–epigenetic regulation of the chromatin and plant signaling–are critically important in all aspects of plant development. It is also easy to envision that the two processes interact. However, studies of this interaction are scarce. Little is known about the effect of plant hormones on plant chromatin structure and the reverse is also true: little is known about the role of chromatin modifications or chromatin modifiers on plant hormone biosynthesis.

Chromatin structure is regulated in part by modification of histones. Through this modification, chromatin function and, therefore, gene regulation are affected. One can regard the higher order of chromatin and nucleosome structure as the final regulatory point in plant hormone signaling pathways for regulation of transcription factors and subsequent gene expression.

At least some effects of plant hormones on gene expression have been shown to depend on specific chromatin modifications, which include histone variants and histone modifications. For example, the expression and accumulation of a “drought-inducible” H1 histone variant (His1) from three tomato species was induced by ABA alone, that is, its induction was unrelated to water deficit (Kahn et al., 1993; Wei and O'Connell, 1996). Other examples come from studies with Arabidopsis. An increase in acetylated histone H3 lysine 14 (H3K14), histone H4 lysine 5 (H4K5), and in tri-methylated H3K4 accompanied ABA activation of the *phaseolin* (*phas*) promoter in transgenic leaves (Ng et al., 2006). Expression of *WRKY70*, a gene that is antagonistically regulated by the SA- and JA-signaling pathways (Li et al., 2004), is controlled by the *ARABIDOPSIS TRITHORAX 1* (*ATX1*) gene, an Arabidopsis homolog of the Drosophila *trithorax*, which activates *WRKY70* expression by establishment of the trimethylation pattern of histone H3 lysine 4 (H3K4me3) of its nucleosomes (Alvarez-Venegas et al., 2007). In rice, down-regulation of *SDG725*, which encodes a histone H3K36 methyltransferase, causes phenotypic defects similar to those described for some brassinosteroid (BR) mutants (Sui et al., 2012), suggesting that *SDG725* depletion results in down-regulation of genes known to be involved in BR signaling, namely, *D11* (*OsDWARF11*), *BU1* (*OsBRASSINOSTEROID UPREGULATED 1*), and *BRI1* (*OsBRASSINOSTEROID INSENSITIVE 1*). Apparently, SDG725-mediated H3K36 methylation regulates BR-related gene expression (Sui et al., 2012). These examples support the assumption that chromatin modification is an important regulator of hormone action on gene expression.

Grain legumes are agronomically important and the common bean (*Phaseolus vulgaris* L.) is the most important grain legume in the human diet (Food and Agricultural Organization of the United Nations, 2014). Grain legumes are recalcitrant to *in vitro* regeneration and *Phaseolus vulgaris* is particularly recalcitrant to *in vitro* induction of somatic embryogenesis and regeneration. Consequently, stable genetic transformation is hard to achieve for this organism, although *P. vulgaris* composite plants, with wild-type (WT) shoots and transgenic hairy roots (derived from *Agrobacterium rhizogenes*-mediated genetic transformation), have been successfully developed (Estrada-Navarrete et al., 2007). However, complete plant genetic transformation of *P. vulgaris* is still elusive. We report here that the creation and establishment of regeneration-competent callus and its transformation are an important first step in the establishment of a stable system of genetic transformation (and possible regeneration) in *P. vulgaris*, necessary for the elucidation of gene function in this important plant. Specifically, we have employed this method to study epigenetic mechanisms that regulate somatic embryogenesis in common bean.

In this study, we were able to generate transgenic embryogenic calli of *P. vulgaris* by particle gun bombardment (Cabrera-Ponce et al., 2015). In these calli, we investigated the role of the gene ortholog to the Drosophila *trithorax* gene, the *Phaseolus vulgaris* Trithorax 1 gene homolog, named *PvTRX1h* (Quiceno-Rico et al., 2012), in somatic embryo development and plant hormone synthesis. When *PvTRX1h* was downregulated by RNA interference (RNAi), pro-embryogenic calli differentiated and formed somatic embryos in abundance and with diverse phenotypes, plant hormone concentrations were altered in ways consistent with the phenotype of the embryos, and *PvTRX1h* downregulation affected the expression of genes involved in auxin biosynthesis. Particularly, our study highlights a clear role of *PvTRX1h* in the regulation of somatic embryogenesis and plant hormone synthesis in plant cells.

We hope that the implemented technology for the generation of transgenic somatic embryos with the potential to regenerate whole transgenic common bean plants will prove useful in the continued development of *P. vulgaris* as a model crop plant and will increase productivity of this important food source.

## Materials and methods

### Plant material

*Phaseolus vulgaris* L. cultivar “Negro Querétaro” was used in this study.

### Vector construction

Construction of the PvTRX1hRiA silencing vector, driving the expression of an antisense sequence from the *PvTRX1h* gene (GenBank locus #JF262910; Phytozome #*Phvul.008G018500*), under control of the ectopic CaMV35S promoter, was created as follows: a 602bp PCR fragment of the *PvTRX1h* cDNA was amplified by using gene-specific forward and reverse primers (PvRX1RiF 5′-tctagagcaaagcatccacataaagg-3′; PvRX1RiR 5′- ggatcccgaaacaatgggaagaatcag-3′; underlined sequences correspond to artificially introduced *Xba*I and *Bam*HI restriction sites, respectively). Next, the pFGC5941 binary vector was digested with *Xba*I and *Bam*HI and the backbone was purified and ligated to the *PvTRX1h* PCR product with the T4 DNA ligase at the *Xba*I and *Bam*HI sites of the pFGC5941 plasmid to generate the PvTRX1hRiA silencing vector, which was used to down-regulate, by RNA interference (RNAi), *PvTRX1h* gene expression.

### Culture media

Osmotic treatment medium: a modified MS medium was used as the basal media and supplemented with Murashige and Skoog micro and macronutrients, 12% (w/v) sucrose (0.368 M), 10 mg/L of 6-Benzylaminopurine (BAP), 40 mg/L adenine free-base, and 2.5 g/L gelrite (Malik and Saxena, 1992; Cabrera-Ponce et al., 2015).

Embryo Induction Medium (EIM): a modified MS medium was used as the basal media and supplemented with Murashige and Skoog micro and macronutrients, 6% (w/v) glucose, 10 mg/L of 6-Benzylaminopurine (BAP), 40 mg/L adenine free base, and 2.5 g/L gelrite (Malik and Saxena, 1992; Cabrera-Ponce et al., 2015).

Regeneration Medium (RM): Murashige and Skoog micro and macronutrients, 0.1 mg/L of kinetin, and 0.4 mg/L of N^6^-(Δ^2^-isopentenyl)adenine (2iP), and 2.5 g/L gelrite (Cabrera-Ponce et al., 2015).

### Zygotic embryo dissection, osmotic treatment, and callus induction

Embryonic axes containing the cotyledonary and apical dome were cultivated for 48 h under osmotic stress in osmotic treatment medium in a growth chamber with a light/dark cycle of 16 h/ 8 h, a photon flux density of 50 μmol m^−2^ s^−1^ provided by fluorescent lamps and one 60-W incandescent bulb, and maintained at 26°C (Cabrera-Ponce et al., 2015). Next, the embryonic axes were transferred to EIM media, kept in a growth chamber under the conditions described above, and used for embryo induction. The first pro-embryogenic mass was obtained 4 weeks after the osmotic shock, mainly from the cotyledonary zone. Pro-embryogenic callus were dissected and transferred to fresh EIM for propagation every 4 weeks (Cabrera-Ponce et al., 2015).

### Callus transformation and selection

Microcarriers for particle gun bombardment were prepared with 100 ng of plasmid DNA. A Bio-Rad PDS-100/He particle delivery system was used (Cabrera-Ponce et al., 1997, 2015). An equimolar mixture of plasmid PvTRX1hRiA and pWRG1515 (Christou et al., 1991) was precipitated onto tungsten microprojectiles of 1.0 μm diameter and delivered onto early globular-stage pro-embryogenic callus that had been sub-cultured for 2–3 months. Ten Petri dishes, each containing 16-20 calli for a total of 2 grams of fresh weight, were bombarded. Control calli were transformed with the pWRG1515 vector only. pWRG1515 contains the *uidA* reporter gene (*gusA*) and the *hptII* gene that confers resistance to hygromycin. Transformed callus were selected in EIM plates containing 50 mg/L hygromycin. After bombardment, calli were sub-cultured every 2 weeks for 3 months in fresh EIM plates containing hygromycin. The transgenic clones maintained their embryogenic capacity, while the construct used in this experiment was stably expressed in the embryogenic callus.

### cDNA synthesis and qRT-PCR analysis

TRIzol reagent (Invitrogen, Carlsbad, CA, U.S.A.) was used to isolate RNA from transgenic callus, control untransformed callus and callus transformed with the empty vector. For qRT-PCR analysis, RNA was treated with DNaseI (Invitrogen, Carlsbad, CA, U.S.A.) to remove genomic DNA. The absence of DNA was confirmed by performing PCR (40 cycles, similar to the real-time PCR program) on the DNaseI-treated RNA using Taq-DNA polymerase. A StepOne® Real-time PCR system (Applied Biosystems, Foster City, CA, U.S.A.) was used for real-time PCR quantifications. qRT-PCR was performed according to the standard SuperScript® II Reverse Transcriptase kit (Invitrogen, Carlsbad, CA, U.S.A.) with the Maxima® SYBR Green/ROX qPCR Master Mix (2x) protocol (Thermo Scientific, Waltham, MA, U.S.A.). A “no DNA” template control was used in each analysis. The results presented are from three independent (*n* = 3) biological replicates (each with eight transgenic calli), and statistical significance was determined with an unpaired two-tailed Student's *t*-test. Each biological replicate was tested by triplicate and data were normalized to the *Actin11* (*PvActin11*) reference gene (PvActin11F 5′-tgcatacgttggtgatgagg-3′, and PvActin11R 5′-agccttggggttaagaggag-3′ (Borges et al., 2012), and to the elongation factor 1-α (*PvEF1*α) reference gene (PvEF1aF 5′-ggtcattggtcatgtcgactctgg-3′, and PvEF1aR 5′- gcacccaggcatacttgaatgacc-3′) (Barraza et al., 2013). The method used to analyze the data from real time PCR experiments corresponds to the relative quantification method, or 2^−ΔΔCT^ method, where the ΔΔCT value = ((CT_1Target_ – CT_1Reference_) – (CT_0Target_ – CT_0Reference_)) (Livak and Schmittgen, 2001). The mean C_T_ values for both the target and internal reference genes were determined and the fold change in the target gene normalized to *PvActin11* and *PvEF1*α and relative to the expression in the control sample. A list of the auxins (IAA), cytokinins (2iP and zeatin), brassinosteroids (epibrassinolide) and abscisic acid (ABA) biosynthetic pathway genes analyzed by q-PCR is shown on Supplementary Table ST1 and a list of the primers used is in Supplementary Table ST2. Amplification of the CaMV35S fragment was performed under the following conditions: 95°C for 10 min, followed by 40 cycles of 95°C/20 s, 54°C/30 s, and 72°C/40 s.

### Frequency of embryogenesis

To determine the frequency of embryogenesis the callus were transferred to fresh EIM for propagation. After 3 weeks, observations on frequency (%) of embryos were recorded. Thirty calli were analyzed for each of the different transgenic clones, as well as for the control callus (non-transformed calli and calli transformed with the empty vector). The number of somatic embryos present per pro-embryogenic mass (PEM) was registered in each clone. Data were analyzed with an unpaired two-tailed Student's *t*-test to compare sample means.

### Histology and optical microscopy

Calli (six per experiment) were embedded in Paraplast after being fixed in 100 mL FAA solution (90 mL 70% ethanol, 5 mL 37% formaldehyde, 5 mL glacial acetic acid), and subjected to a short dehydration ethanol series. Sections of 10 μm were prepared with a microtome (Leica Ultracut R, Vienna, Austria). Samples for optical microscopy were stained with Peryodic acid-Leucobasic Fuchsin-Aniline blue black (Schneider, 1981), analyzed with a light microscope (Motic BA300, Xiamen, China), and photographed with a digital camera (Motic M1000, Xiamen, China).

### Profile of phytohormones by UPLC-ESI-MS

Calli were collected, weighed (200 mg), frozen and ground in liquid nitrogen. Then, the ground tissue was resuspended in 1 mL of methanol:isopropanol:glacial acetic acid (80:19:1), incubated for 48 h at room temperature in darkness and then centrifuged at 10,000 rpm for 15 min, the supernatant was collected and filtered with a 0.22 μm PTFE membrane.

The sample extracts were analyzed using an Ultra Performance Liquid Chromatography Electrospray Mass Spectrometry (UPLC-ESI-MS) system Accela LCQ Fleet Ion trap, Thermo Finnigan, San Jose, CA, USA. The compound mixture was separated on a Hypersil Gold C18 column (50 × 2.1 mm, 1.9 μm particle size). Ten micro-liters of sample were injected. The mobile phase consisted of H_2_O with 0.1% (v/v) formic acid (solvent A) and solvent B was methanol with 0.1% (v/v) formic acid. The column oven temperature was maintained at 35°C, the flow rate was 400 μL/min. The solvent gradient program for free IAA was as follows: 5% B, 0–1 min; 5-95% B, 1–8.9 min; 95% B-95% A, 8.9–9 min; and finally, column re-equilibration for 4 min (9–13 min) with 95% A; while for 2iP, ABA and zeatin the gradient was: 5% B, 0–1 min; 5–60% B, 1–4.9 min; 60% B-95% A, 4.9–5 min; and finally, column re-equilibration for 4 min (5–9 min) with 95% A.

Spectra were acquired in SIM (selected ion monitoring) mode focusing on protonated forms [M + H]: *m/z* 176 (IAA), m/z 204 (2iP), m/z 220 (zeatin) and m/z 265 (ABA) with a *m/z* width of 10, operating in positive mode. The scan time was 500 ms (3 micro-scans).

The ESI source parameters were set as follows: capillary temperature 300°C; capillary voltage 35 V; spray voltage 4.8 kV; tube lens 80 V; nitrogen sheath gas 45 arbitrary units (AU); auxiliary gas 10 AU.

### Histone isolation and western blots

Histones from calli were isolated as previously described (De-La-Peña et al., 2012). In brief, histones were isolated from 1.2 g of tissue from WT and 3-week-old transgenic calli using sulphuric acid extraction of nuclei, followed by acetone precipitation according to established protocols (Jackson et al., 2004). Five micrograms of isolated histones per sample were used for Western blots. The proteins were run on a MINI-PROTEAN SFX, 12% gel (Bio-Rad #456-8043) and transferred to a nitrocellulose membrane (0.45 μm; 24 h, 100 mA constant current, 4°C). The membrane was blocked with 0.5% Tween in Tris-buffered saline (TBS), and re-probed with various antibodies (Merck Millipore, Billerica, MA, USA) as follows: anti-trimethyl-histone H3 [Lys-4] (cat. # 17-614), and anti-trimethyl-histone H3 [Lys-36] (cat. #17-10032). Trimethylated (H3me3/H3) levels were measured and compared to histones isolated from the different transgenic clones and from control calli. The amount of histone H3 for each sample was determined from Western blots using antibodies specific to non-methylated H3 (cat. # 04-928). The Clarity Western ECL Substrate kit from Bio-Rad (cat. # 170-5060), was used to develop the signal (according to the manufacturer's instructions). Both the gel and the membrane were analyzed and documented with the ImageLab software on a Bio-Rad Chemi Doc XR+ imaging system (www.bio-rad.com). Images were recorded every 1.0 s. Signals from bands obtained with methylation-specific antibodies were normalized against the respective histone H3 amounts (measured as the signal intensities of Western blot bands obtained with anti-histone H3 antibodies). Data from four independent measurements (*n* = 4) consistently gave the same results.

## Results

### Induction, cellular differentiation, and somatic embryogenesis in *Phaseolus vulgaris* callus

**Figure 1:**
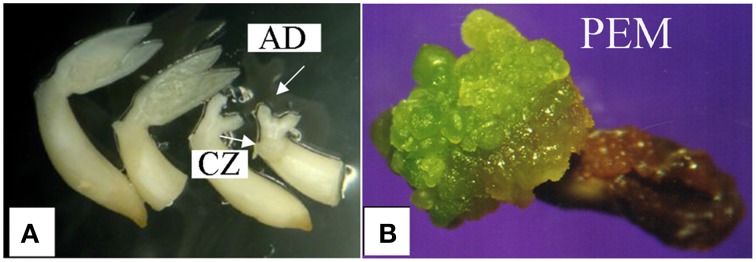
**Osmotic-stress-induced somatic embryogenesis in ***Phaseolus vulgaris*****. **(A)** Zygotic embryos. AD, apical dome; CZ, cotyledonary zone; **(B)** Pro-embryogenic mass (PEM).

To acquire somatic embryos, we employed a recently developed protocol designed for this purpose (Cabrera-Ponce et al., 2015). Common bean embryonic axes from zygotic embryos were cultivated in the osmotic-shock treatment media specified in the protocol (Figure 1A). After incubation, the embryonic axes were transferred to the embryo induction media (EIM). The first pro-embryogenic mass (PEM) was obtained 4 weeks after the osmotic shock, mainly from the cotyledonary zone (Figure 1B), and the pro-embryogenic callus were dissected and transferred to fresh EIM for propagation every 4 weeks.

### Callus transformation and selection

**Figure 2:**
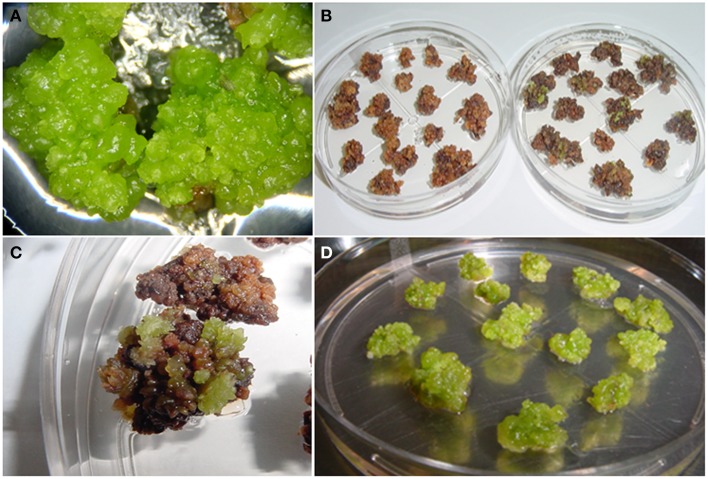
**Transformation and selection of ***P. vulgaris*** calli. (A)** Target embryogenic calli for particle gun bombardment transformation. Bombardment was performed on early globular-stage pro-embryogenic callus that had been sub-cultured for 3 months. Petri dishes containing 16–20 calli were bombarded. **(B)** Hygromycin resistant calli. Transformed callus were selected in EIM plates containing 50 mg/L hygromycin. **(C)** Hygromycin resistant calli, close-up. **(D)** Propagation of hygromycin resistant callus. After bombardment, calli were sub-cultured every 2 weeks for 3 months in fresh EIM plates containing hygromycin.

The *PvTRX1h* gene of common bean is an ortholog of a major histone lysine methyltransferase and a focus of this study. As a first step, we created the PvTRX1hRiA construction, driving the expression of an antisense sequence from the *PvTRX1h* gene. This was used for callus transformation (Supplementary Figure SF1), with the intention that *PvTRX1h* would be down-regulated, by RNA interference (RNAi), in the transformed callus. Transformed calli were sub-cultured in fresh EIM containing hygromycin every 2 weeks for 3 months, until non-bombarded embryogenic callus stopped growing and eventually died. Thus, we monitored stable hygromycin-resistance calli with embryogenic capability for six successive generations following transformation (Figure 2).

**Figure 3:**
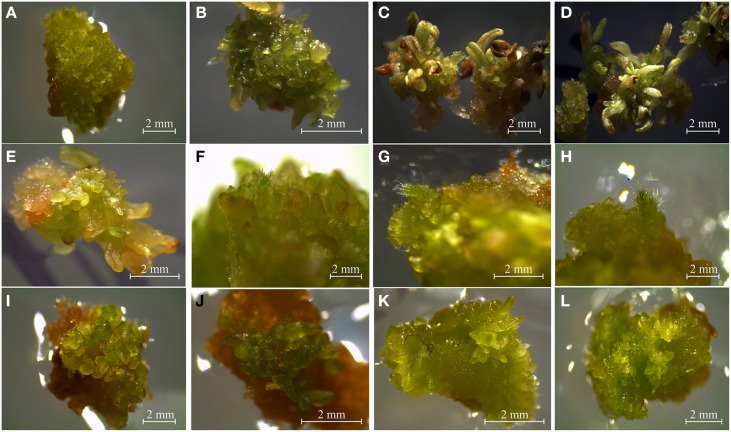
**Phenotypes of pro-embryogenic callus and transgenic embryo formation in 6-week-old common bean. (A)** Control pro-embryogenic callus (friable compact callus). **(B–L)** Transgenic callus transformed with the *PvTRX1h*-RiA silencing vector: **(B–E)** Transgenic Clone 8 (embryonic and shooty callus). **(F–H)** Transgenic Clone 10 (embryonic and rooty callus). **(I,J)** Transgenic Clone 11 (friable callus). **(K,L)** Transgenic Clone 12 (embryonic callus).

After six generations, we had a total of 13 transgenic clones (Figure 2D). However, further maintenance of embryogenic calli for nine of the transgenic clones was not possible. They died after the sixth generation. Thus, 4 out of 13 resistant calli continue dividing for long-term propagation and later characterization (Figure 3). The transgenic calli had quite variable phenotypes and were classified into subgroups based on similarity of their phenotype. According to a recent classification (Ikeuchi et al., 2013), calli with no visible organ regeneration were denoted as friable or compact callus (Figure 3A) and calli that exhibited some degree of organ regeneration were denoted rooty, shooty, or embryonic callus, based upon the type of organs that developed (Figures 3B–L). Clone 8 developed both embryonic and shooty macroscopic structures (Figures 3B–E), clone 10 developed embryonic and rooty macroscopic structures (Figures 3F–H), clone 11 did not develop any organs, (Figures 3I,J), and clone 12 developed embryonic macroscopic structures (Figures 3K,L). Based on these phenotypes, transgenic clones 8, 10, and 12 were selected for further experiments. Clone 11 was not further analyzed because it had no visible organ regeneration.

### Down-regulation of *PvTRX1h* gene in transgenic callus cultured *in vitro*

**Figure 4:**
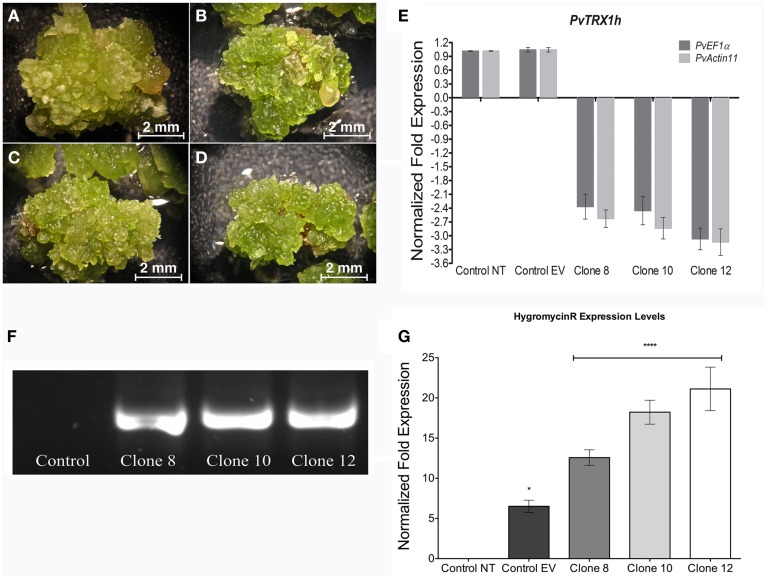
**Phenotypes of 3-week-old transgenic embryogenic calli and down-regulation of ***PvTRX1h*** in transgenic calli. (A)** Control embryogenic callus. **(B)** Transgenic clone 8. **(C)** Transgenic clone 10. **(D)** Transgenic clone 12. **(E**) qRT-PCR analysis of *PvuTRX1h* down-regulation in the transgenic clones. Each biological replicate was tested by triplicate and data were normalized to the *Actin11* (*PvActin11*) and to the *Elongation Factor 1*-α (*PvEF1*α) reference genes. Data represent mean ± SD (*n* = 3 independent experiments) and were analyzed with an unpaired two-tailed Student's *t*-test (^*^P < 0.05, ^****^P < 0.0001). **(F)** PCR amplification of the CaMV35S promoter from genomic DNA to show the transgenic nature of the calli. **(G)** q-RT-PCR analysis of the *hptII* gene, to show the transgenic nature of the calli. Abbreviations: NT, non-transformed callus; EV, callus transformed with empty vector.

We next wished to verify and quantify down-regulation of *PvTRX1h* gene expression in the transgenic clones. We performed qRT-PCR analysis in 3-week-old calli (Figures 4A–D). Transgenic clones 8 and 10 had a 2.4-fold down-regulation and clone 12 had a 3-fold down-regulation of the *PvTRX1h* transcript levels compared to the control, un-transformed calli (and when normalized with both reference genes, *PvActin11* and *PvEF1*α; Figure 4E). The transgenic nature of the calli was further confirmed by performing PCR amplification of the CaMV35S promoter (PvTRX1hRiA construction) from genomic DNA of transgenic callus clones 8, 10, and 12 (Figure 4F), as well as qRT-PCR to test for expression of the *hptII* gene (Figure 4G). This confirmed the transgenic nature of the calli, in agreement with their hygromycin selection or resistance.

### Effects of changes in plant hormones content on callus and shoot formation

The course of acquisition of embryogenic competence by somatic cells involves reprogramming of gene expression patterns in addition to changes in the morphology, physiology, and metabolism of plant cells. Endogenous hormone levels are major factors influencing somatic embryo induction (Fehér et al., 2003).

Thus, in order to study the effect of *PvTRX1h* down-regulation on plant hormone synthesis during somatic embryo formation, we analyzed the concentration of five different plant hormones in the three previously selected transgenic callus clones when they were three 3 s old. The hormones analyzed were indole-3-acetic acid in its free form (or IAA, an auxin), zeatin and N^6^-(Δ^2^-isopentenyl)-adenine (or 2iP; the latter two are cytokinins), ABA (an isoprenoid), and epibrassinolide (a brassinosteroid).

Transgenic calli were cultured on auxin-free EIM medium supplemented with BAP, an inducer of somatic embryogenesis. Next, the plant hormone concentration was determined in the different calli by UPLC-ESI-MS. For IAA the LOD was 20.45 pmol/g fresh weight (FW) and the limit of quatification, LOQ, was 22.69 pmol/g FW; for ABA the LOD was 6.09 pmol/g FW and the LOQ was 10.27 pmol/g FW; for Zeatin the LOD was 22.69 pmol/g FW and the LOQ was 22.69 pmol/g FW; and for 2iP the LOD was 17.51 pmol/g FW and the LOQ was 18.64 pmol/g FW (Supplementary Figure SF2).

**Figure 5:**
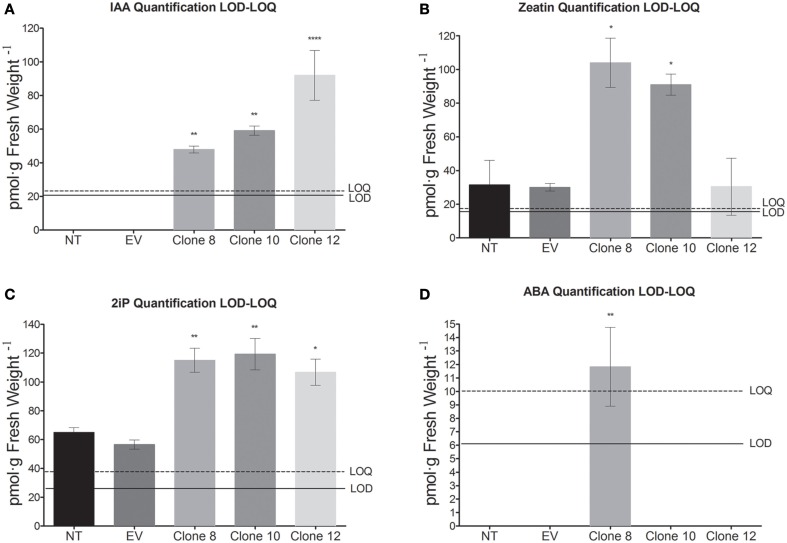
**Plant hormone content determined by UPLC-ESI-MS in 3-week-old ***P. vulgaris*** transgenic callus clones. (A)** IAA, **(B)** zeatin, **(C)** 2iP, **(D)** ABA. Data represent mean ± SD (*n* = 3 independent experiments) and were analyzed with an unpaired two-tailed Student's *t*-test (^*^P < 0.05, ^**^P < 0.01, ^***^P < 0.001, ^****^P < 0.0001). For IAA the limit of detection, LOD, was 20.45 pmol/g fresh weight (FW) and the limit of quatification, LOQ, was 22.69 pmol/g FW; for ABA the LOD was 6.09 pmol/g FW and the LOQ was 10.27 pmol/g FW; for Zeatin the LOD was 22.69 pmol/g FW and the LOQ was 22.69 pmol/g FW; and for 2iP the LOD was 17.51 pmol/g FW and the LOQ was 18.64 pmol/g FW. Abbreviations: NT, non-transformed callus; EV, callus transformed with empty vector.

Compared to the control callus, where the concentration was below the LOD, the IAA concentration in all the transgenic clones was greater than the control callus, ranging from 47 to 92 pmol per gram of fresh weight (Figure 5A). The concentrations of the cytokinins (zeatin and 2iP) are shown in Figures 5B,C. In clones 8 and 10, zeatin concentration increased 232% and 196% compared to the control, respectively, while 2iP increased 76.9% and 83% over the control, respectively. The lowest zeatin concentration was detected in clone 12 (Figure 5B). In this clone, zeatin remained unchanged and 2iP increased as much as 63%, compared to the control. The ABA concentration of clones 10 and 12, as well as the control, were below the LOQ, while there was an increase in ABA concentration in clone 8 (11.8 pmol per gram of fresh weight), as compared to the control (Figure 5D). The epibrassinolide concentration, for all clones, was below the LOD (data not shown).

### Down-regulation of *PvTRX1h* in embryonic callus effects on auxin synthesis

**Figure 6:**
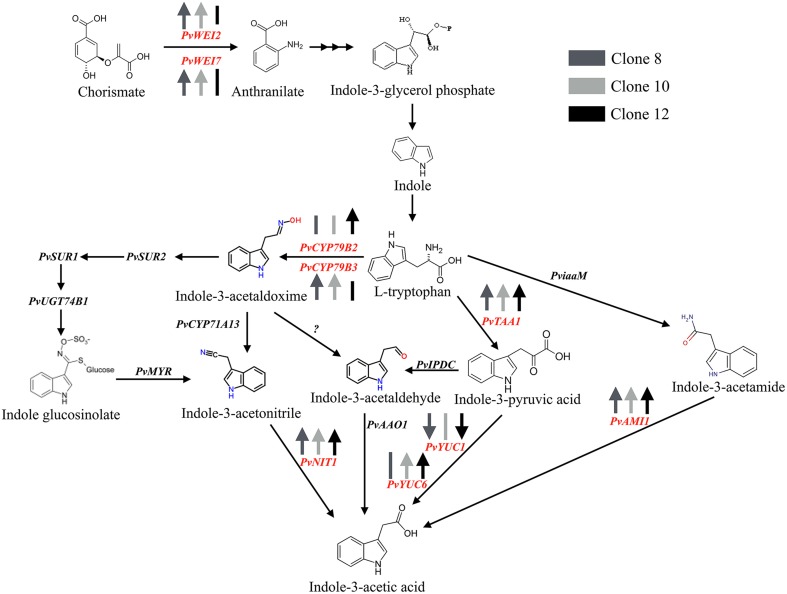
**Potential pathways of IAA biosynthesis in ***Phaseolus vulgaris*****. *De novo* IAA biosynthetic pathways initiate from Trp or Trp precursors. Enzymes analyzed in the common bean in this study are in red. Enzymes in black are those that been identified in Arabidopsis and have orthologs that have been identified in the common bean. Arrows indicate up-regulation or down-regulation of the specified genes in the transgenic callus clones compared to control callus. A vertical bar indicates no change in transcript levels.

**Figure 7:**
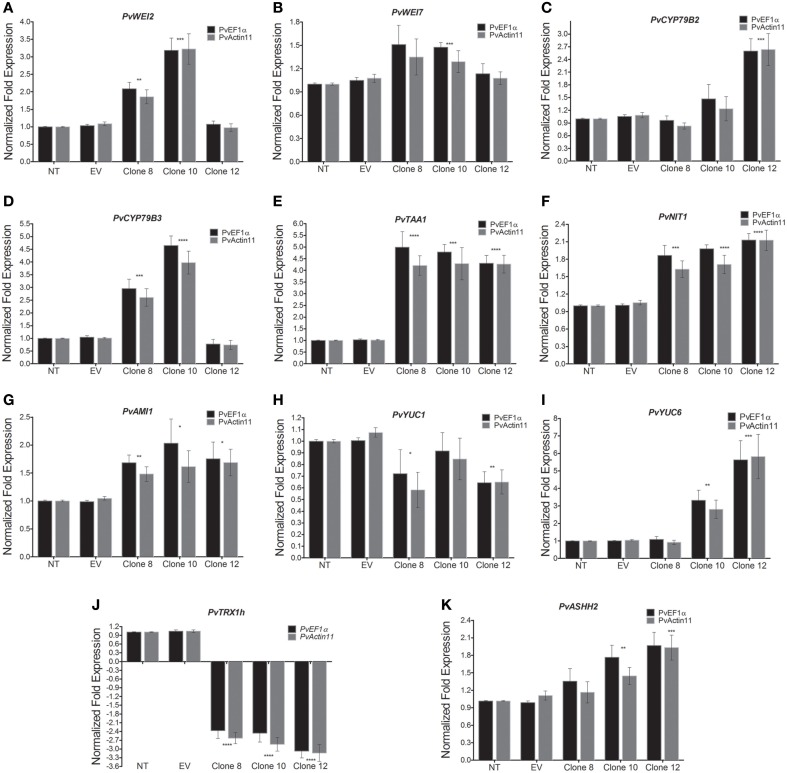
**Transcript levels of some genes involved in IAA biosynthesis as determined by qRT-PCR in 3-week-old callus. (A)** *WEI2* (*ANTHRANILATE SYNTHASE alpha 1, ASA1*), **(B)** *WEI7* (*ANTHRANILATE SYNTHASE beta 1, ASB1*), **(C)** *CYP79B2* (*CYTOCHROME P450 MONOOXYGENASE CYP79B2*), **(D)** *CYP79B3* (*CYTOCHROME P450 MONOOXYGENASE CYP79B3*), **(E)** *TAA1* (*TRYPTOPHAN AMINOTRANSFERASE 1*), **(F)** *NIT1* (*NITRILASE 1*), **(G)** *AMI1* (*INDOLE-3-ACETAMIDE HYDROLASE 1*), **(H)** *YUC1* (*YUCCA1* flavin monooxygenase), **(I)** *YUC6* (*YUCCA6* flavin monooxygenase) **(J)** *PvTRX1h*, **(K)** *PvASHH2h* (*P. vulgaris absent, small or homeotic discs 1 homolog 2*). Data represent mean ± SD (*n* = 3 independent experiments) and were analyzed with an unpaired two-tailed Student's *t*-test (^*^P < 0.05, ^**^P < 0.01, ^***^P < 0.001, ^****^P < 0.0001). Abbreviations: NT, non-transformed callus; EV, callus transformed with empty vector.

Given that the concentration of IAA in its free form increased in all clones in which the *PvTRX1h* gene was down-regulated, we analyzed the transcript levels of some of the genes coding for enzymes directly involved in IAA synthesis. The ones chosen represent the main pathways of tryptophan (Trp)-dependent IAA synthesis (Figures 6, 7; Supplementary Tables ST1, ST2). Specifically, we determined the transcript levels of the gene orthologs to the *Arabidopsis WEI2* (*WEAK ETHYLENE INSENSITIVE2/ANTHRANILATE SYNTHASE* alpha 1, *WEI2/ASA1*) (Stepanova et al., 2005); *WEI7* (*ANTHRANILATE SYNTHASE* beta 1, *WEI7/ASB1*) (Stepanova et al., 2005); *TAA1* (*TRYPTOPHAN AMINOTRANSFERASE 1*) (Stepanova et al., 2008); *AMI1* (*INDOLE-3-ACETAMIDE HYDROLASE 1*) (Pollmann et al., 2003); *CYP79B2* (*CYTOCHROME P450 MONOOXYGENASE CYP79B2*); *CYP79B3* (*CYTOCHROME P450 MONOOXYGENASE CYP79B3*) (Hull et al., 2000; Zhao et al., 2002); *NIT1* (*NITRILASE 1*) (Normanly et al., 1997); and *YUC1* and *YUC6* (*YUCCA1* and *YUCCA6* flavin-containing monooxygenases) (Stepanova et al., 2011; Mano and Nemoto, 2012; Dai et al., 2013) (see Figure 6 for a scheme of IAA synthesis).

We determined the expression levels of these auxin-synthesis-related genes in 3-week-old callus. Compared to the control callus (and when normalized with both reference genes, *PvActin11* and *PvEF1*α), the transcripts of *PvWE12, PvWE17*, and *PvCYP79B3* all increased in clones 8 and 10 (at least doubled, by about 50%, and at least tripled, respectively), but was unchanged in clone 12. In contrast, the transcript level of *CYP79B2* increased by 2.6-fold in clone 12, but was unchanged in the other clones (Figure 7C). The expression levels of *TAA1, NIT1*, and *AMI1* increased in all transgenic callus clones compared to the control (Figures 7E–G). Also, transcripts levels of *YUC1* decreased in transgenic clones 8 and 12, but was unchanged in clone 10 (Figure 7H). However, *YUC6* transcript levels increased from about 2- to 4.8-fold in clones 10 and 12 compared to the control, respectively (Figure 7I); but was unchanged in clone 8.

### Down-regulation of *PvTRX1h* affects the expression of *PvASHH2h*

The concentration of the cytokinins (zeatin and 2iP) and ABA (clone 8) was altered in the transgenic calli compared to the transformed controls (Figure 5), prompting us to measure the expression level of *PvASHH2h*, the gene orthologous to the *Arabidopsis thaliana ABSENT, SMALL or HOMEOTIC DISCS 1 HOMOLOG 2* gene (*ASH1 HOMOLOG 2* or *ASHH2*) in *P. vulgaris*. ASHH2 is a major H3K36 histone lysine methyltransferase (HKMT) in *Arabidopsis* (Xu et al., 2008). This gene has also been shown to be involved in the induction of the jasmonate/ethylene pathway genes (Berr et al., 2010), in the regulation of carotenoid biosynthesis and carotenoid-derived hormones (Cazzonelli et al., 2009), and in the regulation of the expression of BR-related genes (Wang et al., 2014). Expression of *PvASHH2h* increased in clones 8, 10 and 12, as much as 35, 76, and 96% (Figure 7K).

**Figure 8:**
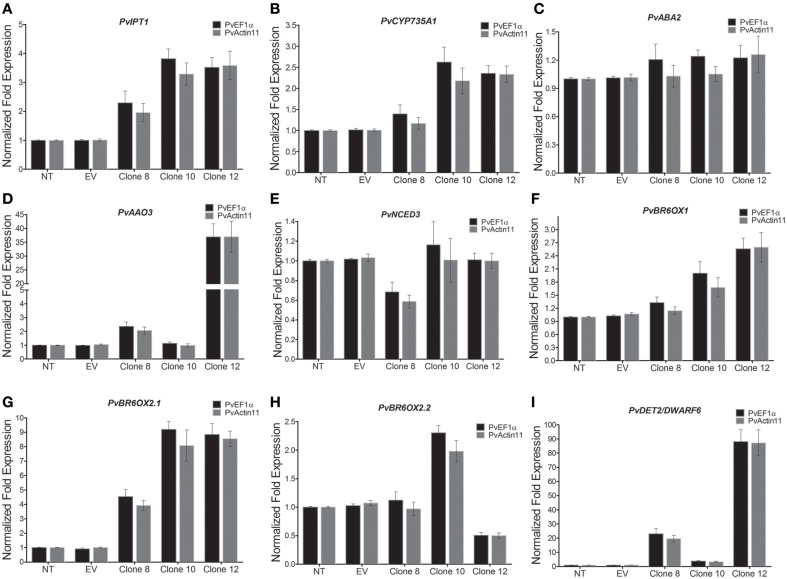
**Transcript levels of some genes involved in zeatin, 2iP, ABA, and BR biosynthesis as determined by qRT-PCR in 3-week-old callus. (A)** *PvIPT1* (*ADENILATE ISOPENTENYLTRANSFERASE 1*), **(B)** *PvCYP735A1* (*CYTOCHROME P450, FAMILY 735, SUBFAMILY A, POLYPEPTIDE 1*), **(C)** *ABA2* (*XANTHOXIN DEHYDROGENASE*), **(D)** *AAO3* (*ABSCISIC ALDEHYDE OXIDASE 3*), **(E)** *NCED3* (*9-CIS-EPOXYCAROTENOID DIOXYGENASE 3*), **(F)** *PvBR6OX1* (*BRASSINOSTEROID-6-OXIDASE 1*), **(G)** *BR6OX2.1* (*BRASSINOSTEROID-6-OXIDASE 2 ISOFORM 1*), **(H)** *BR6OX2.2* (*BRASSINOSTEROID-6-OXIDASE 2 ISOFORM 2*), **(I)** *DET2/DWARF6* (*STEROID REDUCTASE DET2/DWARF6*). Data represent mean ± SD (*n* = 3 independent experiments) and were analyzed with an unpaired two-tailed Student's *t*-test. Abbreviations: NT, non-transformed callus; EV, callus transformed with empty vector.

Next, we determined the transcript levels in the transgenic calli of some genes involved in the synthesis of cytokinins (zeatin and 2iP), ABA, and BR (Figure 8 and Supplementary Table ST1). Specifically, we determined the transcript levels of the gene orthologs to the *Arabidopsis IPT1* (*ADENILATE ISOPENTENYLTRANSFERASE 1*), *CYP735A1* (*CYTOCHROME P450, FAMILY 735, SUBFAMILY A, POLYPEPTIDE 1*), *NCED3* (*9-CIS-EPOXYCAROTENOID DIOXYGENASE 3*), *ABA2* (*XANTHOXIN DEHYDROGENASE*), *AAO3* (*ABSCISIC ALDEHYDE OXIDASE 3*), *DET2/DWARF6* (*STEROID REDUCTASE DET2/DWARF6*), *BR6OX1* (*BRASSINOSTEROID-6-OXIDASE 1*), *BR6OX2.1* (*BRASSINOSTEROID-6-OXIDASE 2 ISOFORM 1*), *BR6OX2.2* (*BRASSINOSTEROID-6-OXIDASE 2 ISOFORM 2*).

Compared to the control callus (and when normalized with both reference genes, *PvActin11* and *PvEF1*α), the expression of *PvIPT1* and *PvCYP735A1*, two genes involved in cytokinin biosynthesis, increased in all transgenic calli, in a straight correlation with the increased concentrations of the cytokinins (zeatin and 2iP) (Figures 8A,B). In contrast, the expression levels of *PvABA2, PvAAO3*, and *PvNCED3*, all involved in ABA biosynthesis, showed dissimilar patterns of expression (Figures 8C–E). *PvABA2* increased by about 0.2-fold in all clones, *PvNCED3* decreased 30% in clone 8, but was unchanged in the other clones, and *PvAAO3* increased by 37-fold in clone 12. Expression of four genes involved in BR biosynthesis (*BR6OX1, BR6OX2.1, BR6OX2.2*, and *DET2/DWARF6*) increased in all transgenic calli (except for *BR6OX2.2* in clone 12), indicative of an up-regulation of BR biosynthesis.

### Changes in histone H3K4 trimethylation patterns as a result of *PvTRX1h* down-regulation

We next wished to examine the effect of down-regulation of *PvTRX1h* on the global patterns of histone methylation. Three-week-old calli were analyzed by Western blots using antibodies against the H3K4 trimethylated isoform (H3K4me3; a histone mark catalyzed by the orthologous gene *ATX1*), as well as for the H3K36me3 mark (a histone mark catalyzed by orthologous gene *ASHH2*).

**Figure 9:**
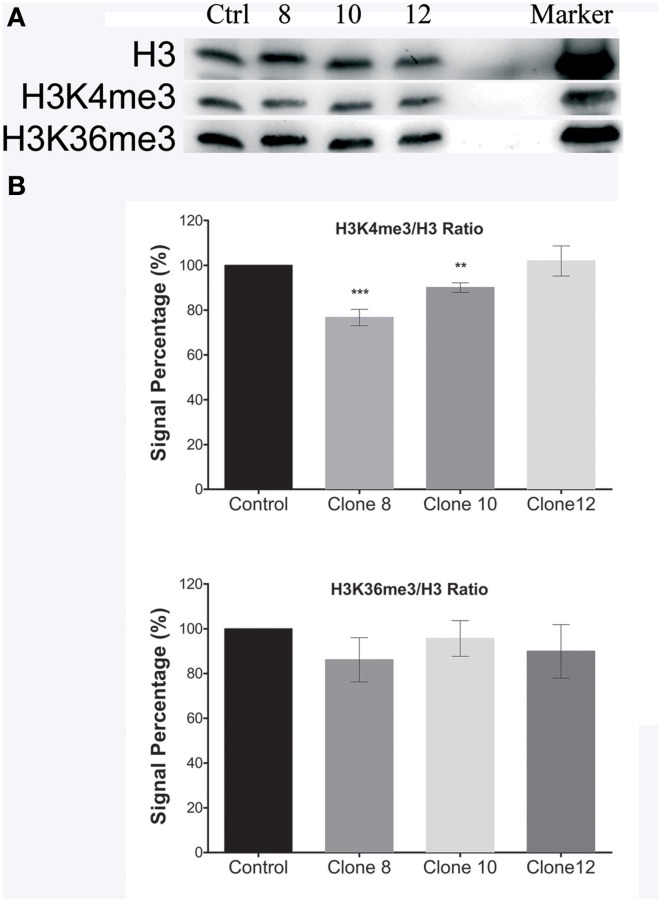
**Histone H3 methylation patterns tested by using Western blot analyses with specific antibodies generated toward each modified histone H3. (A)** Western blot analysis shows the amount of histone methylation in the transgenic clones. **(B)** Bar graphs showing quantification of the histone methylation (H3K4me3 and H3K36me3) in the transgenic clones. Total histones extracted from 3-week-old non-transformed (control) and transgenic calli samples were probed with antibodies specific for tri-methylated K4/H3 and K36/H3 in Western blots. Subsequent to the hybridization, membranes were stripped off and reprobed with antibodies specific for non-modified histone H3. The levels of histone H3-tail methylation, defined as the ratio of mK/H3-to-H3 intensity signals, were taken as 100%. Data represent mean ± SD (*n* = 4 independent experiments) and were analyzed with an unpaired two-tailed Student's *t*-test (^**^P < 0.01, ^***^P < 0.001).

We observed changes in global H3K4me3 methylation patterns in the different transgenic clones that are related to *PvTRX1h* down-regulation, to the *PvASHH2h* transcript levels, and to the plant hormone concentration. In clones 8 and 10, with a 2.4-fold down-regulation of the *PvTRX1h* gene, the global H3K4me3 mark decreased (22 and 10%, respectively; Figure 9), but *PvASHH2h* expression increased (35 and 76%, respectively), accompanied by an increase in of IAA (in its free form) and cytokinins compared to the control. In transgenic clone 12, with 3-fold down-regulation of *PvTRX1h*, the global H3K4me3 mark increased 6%, *PvASHH2h* expression increased 96.7%, and IAA and 2iP increased compared to the control. However, even though *PvASHH2h* expression increased, the traceable changes in the global H3K36me3 methylation patterns are not statistically significant (Figure 9). This could be due to, for example, the level of methylation (mono-, di-, tri-) imparted and associated with the transcription of active euchromatin, as well as the transcriptional repression associated with H3K36 methylation, through modulating H3K36 (mono-, di-, tri-) methylation levels (Wagner and Carpenter, 2012).

### Morphology and histology of transgenic embryonic callus

**Figure 10:**
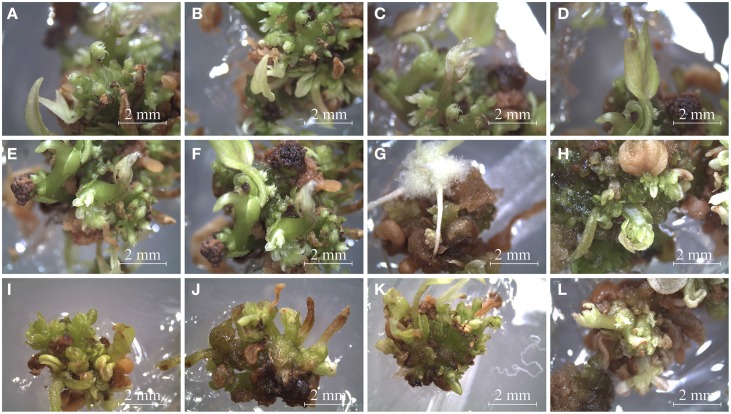
**Differentiation of embryogenic calli from 10-week-old transgenic clone 8, cultured on regeneration media**. **(A–F):** callus No. 1, **(G)**: callus No. 2, **(H)**: callus No. 3, **(I–K)**: calli No. 4 and 5.

**Figure 11:**
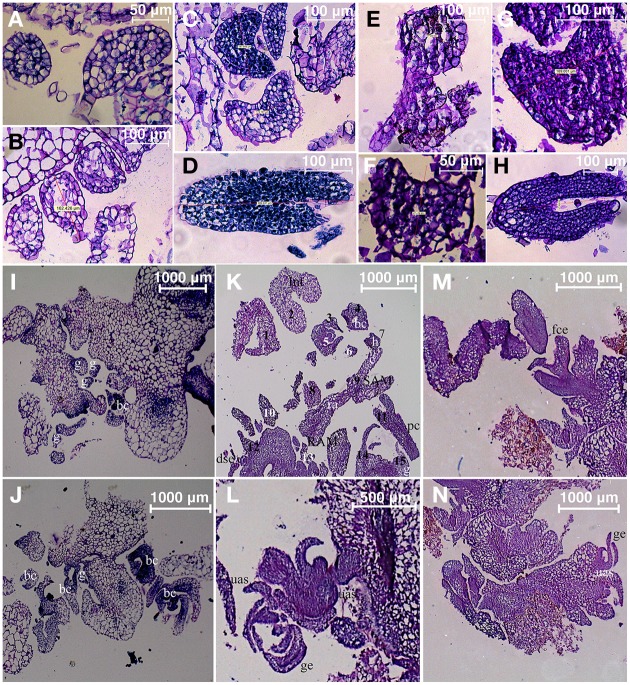
**Histological sections from 10-week-old transgenic clone 8**. Ontogeny of somatic embryos. **(A–D)** Micrographs of histological sections of control callus: **(A,B)** Globular stage embryo, **(C)** heart-shaped stage embryo, **(D)** torpedo stage embryo. **(E–H)** Micrographs of histological sections of transgenic clone 8: **(E)** globular stage embryo, **(F,G)** heart-shaped stage embryo, **(H)** torpedo stage embryo. **(I,J)** Micrographs of histological sections of control callus. **(K–N)** Micrographs of histological sections of transgenic callus clone 8, showing multiple shoot meristems: **(K)** longitudinal section of somatic embryos (numbering corresponds to number of embryos per section), **(L)** longitudinal section of well-developed shoot formed from callus, **(M,N)** longitudinal sections of somatic embryos. (g, globular; t, torpedo; Int, intermediate; bc, bent cotyledon; h, heart; c, cotyledonary; dse, differentiation of secondary embryos; SAM, shoot apical meristem; RAM, root apical meristem; ra, root axis; pc, procambium; fce, fasciated cotyledonary embryo; ge, germinated embryo. uas, unipolar adventitious shoot).

**Figure 12:**
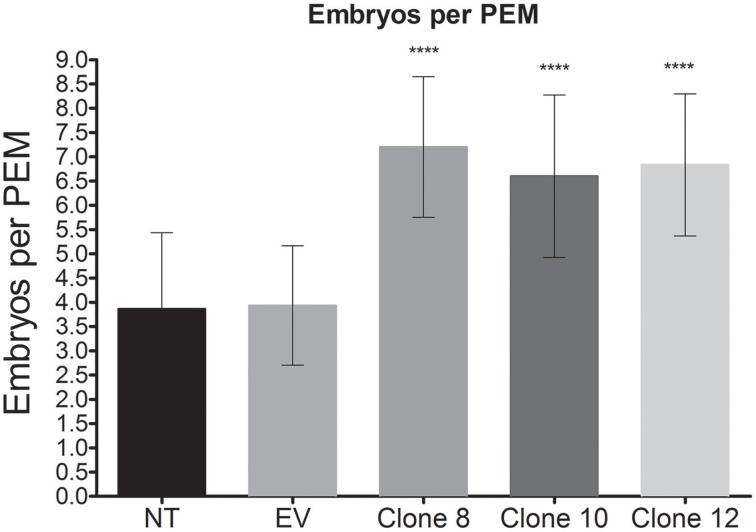
**Frequency of embryogenesis**. Thirty calli were analyzed for each clone, as well as for the control calli (non-transformed calli and calli transformed with the empty vector). The number of somatic embryos present per pro-embryogenic mass (PEM) was registered for each clone. Data represent mean ± SD (*n* = 30 independent callus) and were analyzed with an unpaired two-tailed Student's *t*-test (^****^P < 0.0001). Abbreviations: PEM, pro-embyogenic mass; NT, non-transformed callus; EV, callus transformed with empty vector.

The next endeavor was to characterize the developmental stages of common-bean somatic embryos and the histology of the various structures and tissues at the stages (shown in Figure 10). For this purpose, and taking into account the inherent variation of transgene expression in the different transformants, clone 8 was chosen because it evidenced several developmental stages of somatic embryos in the same embryogenic callus (Figures 3, 10, 11). This clone also had an interesting phenotype, with embryonic, shooty, and rooty macroscopic structures and was the most prolific of the clones with respect to somatic embryogenesis (Figure 12). That is, 3 weeks after the callus were transferred to fresh EIM, clone 8 had 7.2 somatic embryos present per pro-embryogenic mass (PEM), clone 10 had 6.6 and clone 12 had 6.8 somatic embryos present per PEM, respectively. Whereas the non-transformed calli and those calli transformed with the empty vector had 3.8 and 3.9 somatic embryos present per PEM.

Histological examination of clone 8 (ontogeny of somatic embryogenesis; Figure 11), revealed that early-stage calli were not masses of unorganized cells, but had highly organized early-stage structures, specifically: globular (Figures 11A,B,E), heart (Figures 11C,F,G), intermediate, or torpedo early stages (Figures 10D,H), reminiscent of dicotyledonous embryos (Figures 11J,K). The globular, heart, and torpedo embryos of this clone and the WT's were of similar size. As many as 15 somatic embryos per 10-μm section of callus could be discerned under the microscope (Figure 11).

Figure 11 shows an embryo at late globular stage with a suspensor-like structure, in which some rows of cells appear to support a connection between the globular embryo and the parental tissue (Figures 11A,B,E). According to Williams and Maheswaran (1986), embryos attached to parental tissue by a suspensor-like structure may originate from a single cell.

The embryos progress was monitored. They developed normally from globular to heart-shaped, torpedo, then cotyledonary stages. Typical structures of WT mature embryo stages (late torpedo to cotyledonary stage) were apparent: cotyledons, apical meristems, procambium tissue, shoot primordia, and root axes (Figures 11J–L). In addition, we observed *de novo* meristemoid structures and unipolar adventitious shoots that emerged from the parental tissue. (Figure 11L). Some abnormal histo-differentiated embryos were observed. For example, fused embryos developed into what looked like fasciated-like cotyledonary embryonic structures (Figure 11M) that seemed to result from fusion of early-stage globular embryos (Dos Santos et al., 2006).

At least for clone 8, the decrease in global H3K4me3, the small increase in the *PvASHH2h* expression, and the increase in the concentration of all four hormones tested may indicate that the level of down regulation of *PvTRX1h*, associated with such changes, are favorable conditions to initiate callus differentiation.

## Discussion

The goals of this study were 2-fold: first, we have endeavored to develop regeneration-competent callus in common bean and second, we have studied the regulation of somatic embryogenesis in this crop plant, choosing to focus on epigenetic regulation. Common bean is an important food and feed crop worldwide and there are ongoing, major efforts to improve it. These efforts, at present, are mainly limited to conventional breeding practices, as this crop is recalcitrant to both induction of somatic embryogenesis and transformation, and is difficult to regenerate. Consequently, stable genetic transformation is hard to achieve for this organism. Thus, the development of regeneration-competent callus and its successful transformation would be a valuable first step toward establishing an efficient plant regeneration system and genetic transformation in *P. vulgaris*. Although regeneration-competent callus have been obtained from pedicels of two genotypes of *P. vulgaris* (Mohamed et al., 1993) and, through a similar approach, regeneration was achieved in tepary bean (*P. acutifolius*) (Dillen et al., 1996), the protocol is considerably less reproducible and efficient than reported (Zambre et al., 1998).

The potential for somatic embryogenesis varies with plant species and among genotypes within a species (Deo et al., 2010) and, therefore, would seem to be related to gene expression and not to the absence of the relevant genes. One possible mechanism of regulation, chromatin remodeling, has two major roles during the early stages of somatic embryogenesis. Differentiation (associated with the first phase of chromatin de-condensation) requires unfolding of the supercoiled chromatin structure, allowing expression of genes previously inactivated by heterochromatinization. Subsequent chromatin remodeling results in the specific activation of a set of genes required for embryonic development (Fehér et al., 2003; Solís-Ramos et al., 2012).

We consider that the phenotypic variation observed in our transgenic lines, which ranged from highly embryogenic to minimally embryogenic or recalcitrant, was due to differential expression of *PvTRX1h*, specifically, down-regulation, in association with the changes in plant hormone concentration reported here, although the known variation of transgene expression in transformants should not be discounted. Furthermore, the alterations in the expression of the genes analyzed here, specifically, the genes coding for enzymes in the plant hormones biosynthetic pathways, *PvASHH2h*, and the many other unidentified genes important at various stages of somatic embryogenesis, are presumably an indirect result of *PvTRX1h* down-regulation, since the orthologs to PvTRX1h are involved in the establishment of the trimethylation pattern of histone H3 lysine 4 (H3K4me3), a mark related to gene activation.

Since the pioneering work by Skoog and Miller (1957), it has been understood that the balance between auxins and cytokinins is reflected in the state of cell differentiation and dedifferentiation. A high auxin to cytokinin ratio induces root regeneration, whereas a low ratio promotes shoot induction, suggestive of auxin-cytokinin crosstalk during *in vitro* organogenesis, although the molecular mechanism of such interaction in the *in vitro* meristem formation is unknown (Su et al., 2011). The observation that the somatic embryos of the different clones in this study had a variety of developmental stages leads us to speculate that down-regulation of *PvTRX1h* in the transgenic callus influenced the concentrations of plant hormones, particularly auxins (free IAA) and cytokinins.

This speculation was directly supported with plant hormone measurements in the *PvTRX1h*-RNAi transgenic calli and the non-transformed callus. The IAA content of all three transgenic calli clones was greater than the control line and we hypothesize that the changes in plant hormones concentration, particularly the auxin to cytokinin ratio and the increase in free IAA content, caused the developmental effects observed, most striking in clone 8 (Figures 3, 10). We stress the importance of auxin partly because of its well-known effects on nearly every aspect of plant growth and development (Woodward and Bartel, 2005) as well as our finding that IAA, in its free form, was the only plant hormone that could be quantified in excess in all clones tested.

The quantification of the plant hormones analyzed leads us to hypothesize that there was crosstalk between the different hormones, especially between IAA and cytokinins. Also, this crosstalk appears to be a result of the different degrees of *PvTRX1h* gene down-regulation in the respective calli (Figure 4) and may be related to the different phenotypes observed (Figures 3, 10). However, the molecular mechanism of such hormone interaction in calli remains unknown.

The increase of free IAA content in all the *PvTRX1h*-RNAi clones led us to analyze the expression patterns of the genes involved in its biosynthesis (Figures 6, 7). Two major pathways for IAA biosynthesis have been proposed: the Trp-dependent and Trp-independent. The Trp-independent path branches from the L-Trp biosynthetic pathway at steps involving indole or indole-3-glycerol phosphate, although the pathway has not been completely elucidated. On the other hand, the Trp-dependent pathways have been carefully characterized and we analyzed the transcript levels of some genes involved in these pathways.

Four main paths for the Trp-dependent IAA syntheses have been described: the indole-3-acetaldoxime (IAOx), tryptamine (TAM), indole-3-acetamide (IAM), and the indole-3-pyruvic acid (IPA) pathways (Stepanova et al., 2005; Su et al., 2011) (Figure 7). *WEI2* and *WEI7* code for the alpha- and beta-subunits, respectively, of a rate-limiting enzyme for Trp biosynthesis, anthranilate synthase (Stepanova et al., 2005), which catalyzes the conversion of chorismate to anthranilate. Transcriptional induction of *WEI2* and *WEI7* should, therefore, enhance auxin biosynthesis, as proposed by Stepanova (Stepanova et al., 2005), and as we have shown here for the transgenic calli. The two P450 monooxygenases, CYP79B2 and CYP79B3, oxidize Trp into indole-3-acetaldoxime (IAOx) *in vitro*. We suggest then, that the differential and specific expression levels of these two genes in the different transgenic clones reflect the redundancy within IAA biosynthetic pathways, as they might compensate each other during IAOx synthesis (Zhao et al., 2002). Whereas the YUCCA (YUC1 and YUC6) flavin-containing monooxygenases (FMOs) catalyze a rate-limiting step in auxin biosynthesis; they convert indole-3-pyruvate (IPA) to indole-3-acetate (IAA).

*WEI2, WEI7*, and *CYP79B3* were up-regulated in clones 8 and 10, while clones 10 and 12 showed specific up-regulation of *CYP79B2* (Figure 7). The *TAA1, AMI1*, and *NIT1* genes were up-regulated in all three clones. *YUC6* was up-regulated in clones 10 and 12, while *YUC1* was down-regulated in all clones. From these results, we hypothesize that *PvTRX1h* has an indirect effect on the synthesis of IAA. It appears that *PvTRX1h*, by regulating chromatin structure in developmental transitions, is able to regulate the expression of genes involved in IAA biosynthesis, most likely through the activation or repression of an unknown intermediary effector. Altogether, the changes in the transcript levels for the genes involved in IAA biosynthesis, as shown on Figures 6, 7, led to an overproduction of free IAA in the transgenic clones.

We were also interested in the expression levels of *PvASHH2h*, an ortholog to a major histone lysine methyltransferase in Arabidopsis able to methylate lysine 4 and 36 of histone H3 (H3K4 and H3K36; Xu et al., 2008). Like *PvTRX1h*, this gene is implicated in plant hormone biosynthesis. We observed an inverse correlation between the up-regulation of this gene and the down-regulation of *PvTRX1h* (Figures 7H,I). Transgenic callus clone 12 had the greatest down-regulation of *PvTRX1* and, in turn, the highest expression of *PvASHH2h*, along with the greatest concentration of IAA (and to some extent 2iP). Clone 10 had the second highest expression of *PvASHH2h*, while clone 8 was lower. These results could be consistent with two sets of observations relating to these genes in *Arabidopsis*. First, the *ARABIDOPSIS TRITHORAX 1* (*ATX1*) gene, which is orthologous to *PvTRX1h*, participates in cell proliferation and cell patterning processes in the root apical meristem independently of auxin and the developmental abnormalities seen in *atx1-1* roots are unrelated to auxin response gradients (Napsucialy-Mendivil et al., 2014). Second, ASHH2 (or SDG8) is involved in the activation of a subset of genes within the JA/ET signaling defense pathway (Berr et al., 2010) and in BR-regulated gene expression (Wang et al., 2014), and the *ashh2* loss-of-function mutant displays a reduced growth phenotype with compromised JA/ET and BR responses (Berr et al., 2010; Wang et al., 2014). These suggest that the two trithorax-Group (TrxG) histone methyltransferases carry out opposite non-redundant functions in callus (evidence provided in this study), roots, and plant development in general (Alvarez-Venegas et al., 2006; Xu et al., 2008; Berr et al., 2010; Napsucialy-Mendivil et al., 2014; Wang et al., 2014).

We also assessed the global patterns of histone methylation by Western blots, using antibodies against H3K4me3, as well as against the H3K36me3 mark. As noted earlier, greater *PvTRX1h* down-regulation resulted in higher *PvASHH2h* expression, and this resulted in re-establishment of the global histone H3K4me3 methylation levels (mainly for clone 12). This might be indicative of crosstalk between these two histone methyltransferases. We hypothesize that the changes in the global H3K4me3 mark in the transgenic clones, directly associated with the deposition of the same mark (as well as the H3K36me3 mark) by another specific histone methyltransferases (*PvASHH2h*), are involved in the synthesis of plant hormones and, in the case of IAA, are related to the changes in transcript levels of the different genes involved in its synthesis.

The down-regulation of *PvTRX1h* permitted us to generate embryogenic calli. These calli overproduced several compounds, most notably IAA and cytokinins. Hormonal signaling pathways interact at the level of gene expression. For example, cytokinin and auxin are antagonistic during lateral root initiation. Cytokinins perturb the expression of *PIN* genes in lateral root founder cells, inhibiting the formation of an auxin gradient that is necessary for lateral root initiation (Laplaze et al., 2007). Also, target genes repressed by auxin are also repressed by brassinosteroids, and genes induced by auxin are induced by brassinosteroids, indicative of coordination between the signaling pathways (Santner and Estelle, 2009).

The increased levels of both free IAA and cytokinins in the *PvTRX1h*-RNAi transgenic calli may also be related to the unforeseen over-expression of the *PvASHH2h* gene. For example, down-regulation in rice of the *SDG725* gene, orthologous to *ASHH2*, which codes for a histone H3 lysine 36 methyltransferase, produces plants with phenotypes that are very similar to those of BR-deficient mutants (Sui et al., 2012). It is noteworthy that several BR biosynthesis-related genes are down-regulated in *SDG725* RNAi mutants (Sui et al., 2012). Accordingly, the up-regulation of the *PvASHH2h* gene in the *PvTRX1h* RNAi transgenic calli, and the up-regulation of several genes involved in the biosynthesis of BR (Figure 9) suggests that *PvASHH2h* has an active role in the regulation of BR-related genes and BR biosynthesis in common bean calli, as it does in rice and Arabidopsis (Normanly et al., 1997; Sui et al., 2012), although the epibrassinolide concentration was below the LOD in all transgenic calli. This also supports our contention that the *PvTRX1h* and *PvASHH2h* genes carry out opposite non-redundant functions in callus, roots, and in plant development in general. Altogether, the free IAA content enhancement and the variation in cytokinins concentration for the clones analyzed support indirect regulation by PvTRX1h of biosynthesis of some of the plant hormones and somatic embryo development.

## Conclusions

Down-regulation of the *PvTRX1h* gene in pro-embryogenic calli of *P. vulgaris* had multiple effects. Transgenic pro-embryogenic calli were able to differentiate and form somatic embryos with diverse phenotypes, an overproduction of somatic embryos was achieved in the transgenic clones, the concentration of different plant hormones was altered, and *PvTRX1h* appeared to regulate the expression of genes involved in auxin biosynthesis. These observations suggest that *PvTRX1h* regulates somatic embryogenesis and plant hormone synthesis. In addition, *PvTRX1h* and *PvASHH2h* carry out opposite non-redundant functions in embryogenesis, indicative of crosstalk among histone methyltransferases and plant hormone signaling. Furthermore, our results indicate that epigenetic changes such as histone methylation have an active role in the regulation of plant hormone biosynthesis in common bean calli, as has been shown in rice and Arabidopsis for BR-related genes and BR biosynthesis. New approaches of this kind and the development of new technologies, particularly regeneration of common bean plants, will undoubtedly increase our knowledge of the crosstalk among histone methyltransferases, plant hormone signaling, and gene regulation of somatic embryogenesis.

## Author contributions

RA provided the idea of the work. RA, AB, JC, and RW designed the experiments. AB conducted the histological analysis, Western blots, RT-PCR, qRT-PCR, and sample preparation for UPLC-ESI-MS experiments. JC conducted the callus transformation and selection. RG, AB, and RW performed the UPLC-ESI-MS analysis. FL created the PvTRX1hRiA silencing vector. RA, AB, and RW participated in the interpretation of results and critically reviewed the manuscript. RA wrote the paper. All authors read and approved the final manuscript.

### Conflict of interest statement

The authors declare that the research was conducted in the absence of any commercial or financial relationships that could be construed as a potential conflict of interest.

## Supplementary material

Supplementary Figure SF1 **Map of the PvTRX1hRiA construction**. Construction of the PvTRX1hRiA silencing vector, driving the expression of an antisense sequence from the *PvTRX1h* gene, under control of the ectopic CaMV35S promoter.

Supplementary Figure SF2 **Establishment of the limit of detection (LOD) and the limit of quantification (LOQ) based in the signal intensity percentage, derived from the UPLC-MS intensity data, of transgenic callus clones compared with control callus of the different plant hormones analyzed**. In the cases where the signal was below the LOD and LOQ, “spiking” determinations were performed. **(A)** IAA content was determined for transgenic callus clones since IAA signal intensity was above the LOD (20.45 pmol/g FW)and LOQ (22.69 pmol/g FW), whereas in control callus IAA signal intensity was below the LOD and LOQ. **(B)** ABA content was determined only for transgenic callus clone 8 since ABA signal intensity was above LOQ (10.27 pmol/g FW); however in transgenic callus clones 10 and 12, and control calli, ABA signal intensity were above the LOD (6.09 pmol/g FW) but below LOQ (10.27 pmol/g FW). **(C)** Zeatin content was determined for all transgenic callus clones and control callus since Zeatin signal intensity for all samples analyzed were above LOD (22.69 pmol/g FW) and LOQ (22.69 pmol/g FW). **(D)** 2iP content was determined for all transgenic calli clones and control calli since 2iP signal intensity for all samples analyzed were above the LOD (17.51 pmol/g FW) and LOQ (18.64 pmol/g FW).

Supplementary Table ST1 **List of the auxins (IAA), abscisic acid (ABA), cytokinins (2-iP and zeatin), and brassinosteroids (epibrassinolide) biosynthetic pathway genes analyzed by qPCR**.

Supplementary Table ST2 **Primer list used for determination of expression levels by q-PCR and presence of the CaMV35s promoter**.
