# Supplementary figures and images for: The Phaseolus vulgaris PvTRX1h gene regulates plant hormone biosynthesis in embryogenic callus from common bean
Source: Front Plant Sci. 2015 Jul 28;6:577. doi: 10.3389/fpls.2015.00577 (PMC4516878; doi:10.3389/fpls.2015.00577)

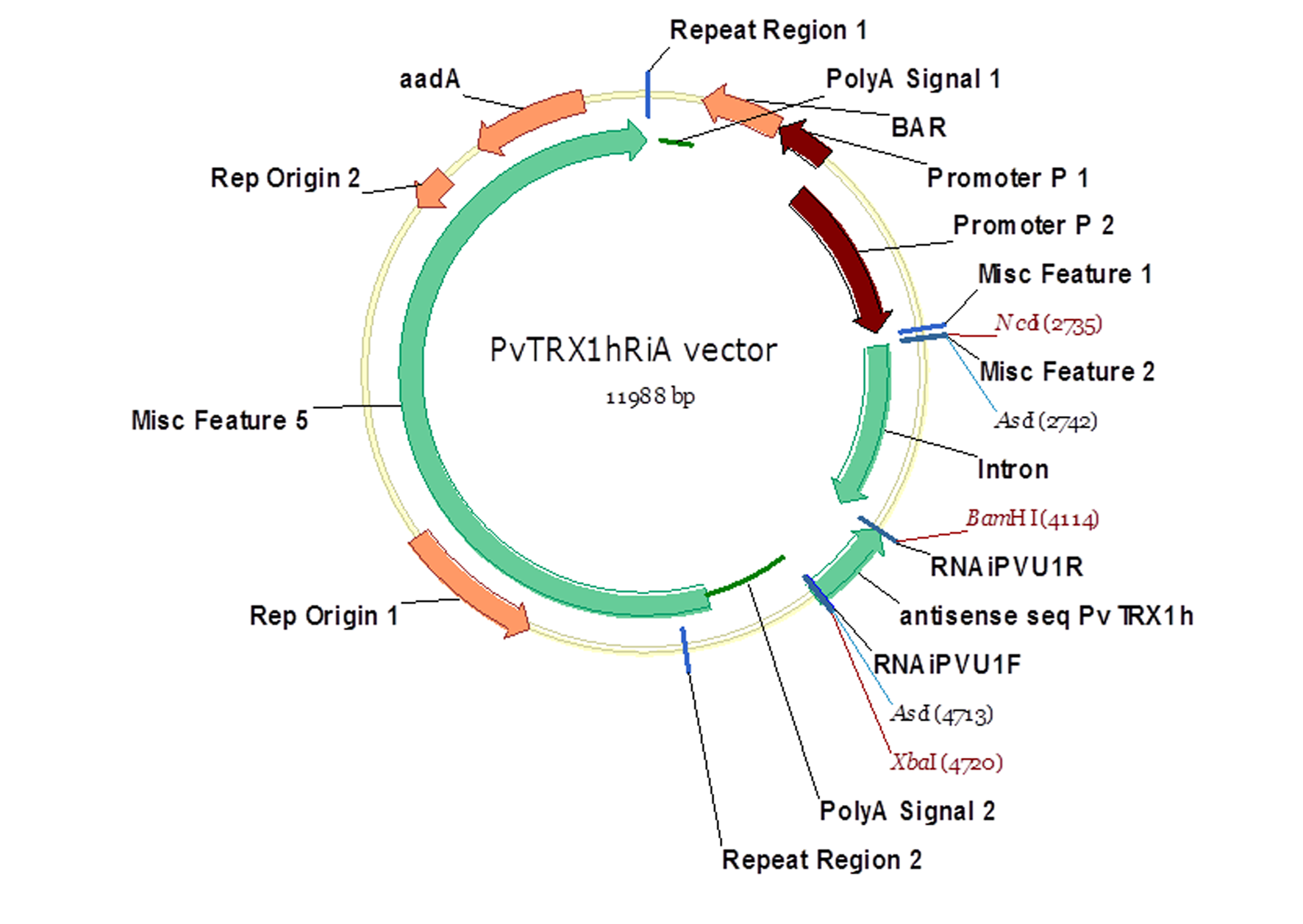

Supplement: Supplementary Figure SF1 — Map of the PvTRX1hRiA construction. Construction of the PvTRX1hRiA silencing vector, driving the expression of an antisense sequence from the PvTRX1h gene, under control of the ectopic CaMV35S promoter. [file Image1.TIF]

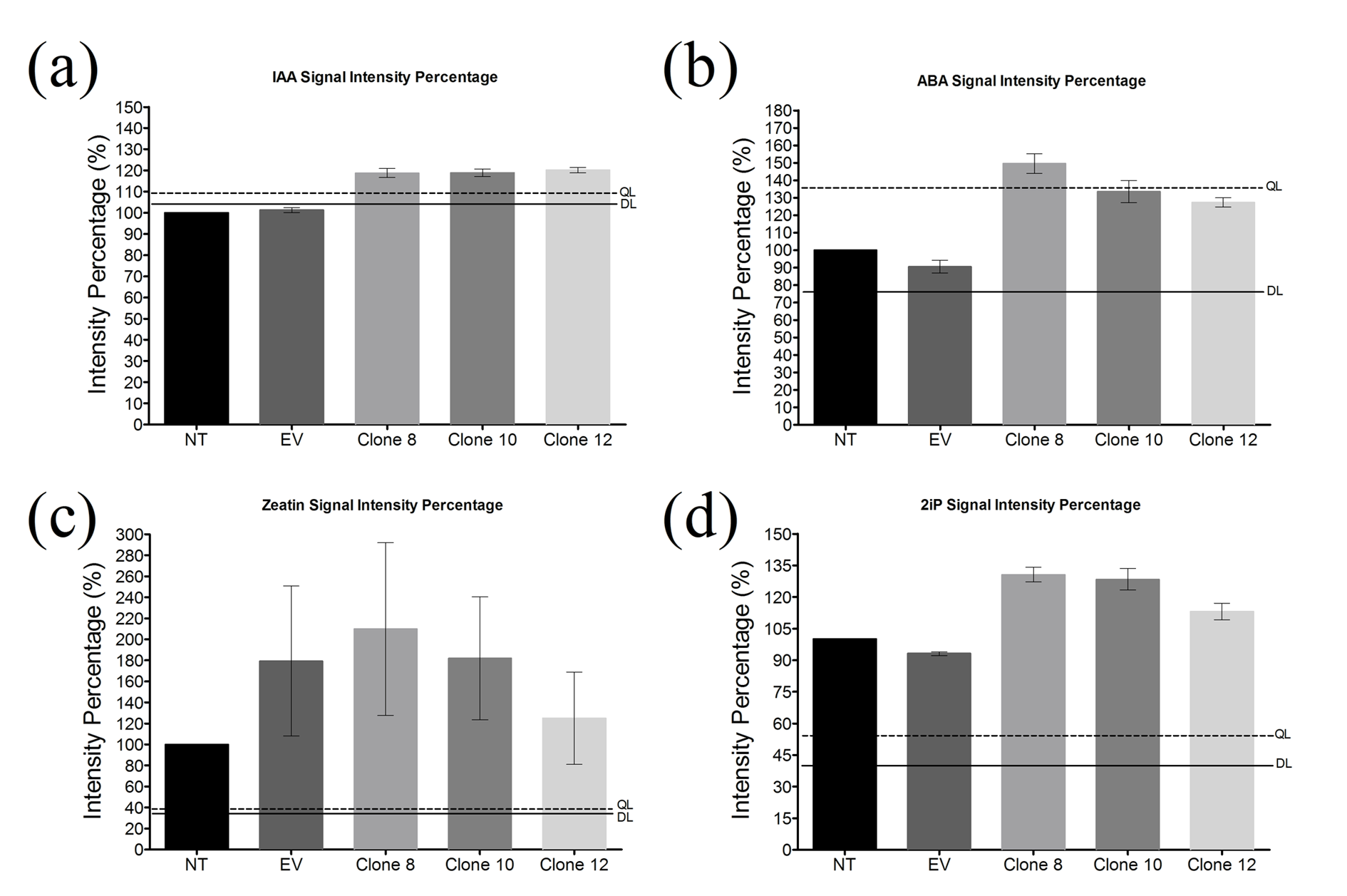

Supplement: Supplementary Figure SF2 — Establishment of the limit of detection (LOD) and the limit of quantification (LOQ) based in the signal intensity percentage, derived from the UPLC-MS intensity data, of transgenic callus clones compared with control callus of the different plant hormones analyzed. In the cases where the signal was below the LOD and LOQ, “spiking” determinations were performed. (A) IAA content was determined for transgenic callus clones since IAA signal intensity was above the LOD (20.45 pmol/g FW)and LOQ (22.69 pmol/g FW), whereas in control callus IAA signal intensity was below the LOD and LOQ. (B) ABA content was determined only for transgenic callus clone 8 since ABA signal intensity was above LOQ (10.27 pmol/g FW); however in transgenic callus clones 10 and 12, and control calli, ABA signal intensity were above the LOD (6.09 pmol/g FW) but below LOQ (10.27 pmol/g FW). (C) Zeatin content was determined for all transgenic callus clones and control callus since Zeatin signal intensity for all samples analyzed were above LOD (22.69 pmol/g FW) and LOQ (22.69 pmol/g FW). (D) 2iP content was determined for all transgenic calli clones and control calli since 2iP signal intensity for all samples analyzed were above the LOD (17.51 pmol/g FW) and LOQ (18.64 pmol/g FW). [file Image2.TIF]
